# Patient experiences of WALANT for outpatient upper limb surgery: A cross-sectional study of perioperative pain and anxiety

**DOI:** 10.4102/jcmsa.v3i1.184

**Published:** 2025-06-13

**Authors:** Reshlan Govender, Antoine G.L. Rocher, Mario T. Rankin, Khangelani Sondovana, Simphiwe C. Phenyane, Megan O’Connor

**Affiliations:** 1 Department of Orthopaedics, School of Clinical Medicine, Faculty of Health Sciences, University of KwaZulu-Natal, Durban, South Africa; 2 School of Clinical Medicine, Faculty of Health Sciences, University of KwaZulu-Natal, Durban, South Africa

**Keywords:** WALANT, patient experience, VAS, anxiety, upper limb surgery, outpatient surgery, orthopaedic surgery, hand surgery

## Abstract

**Background:**

Demands on South African operating theatres have led to more outpatient upper limb surgery being performed under wide awake local anaesthesia no tourniquet (WALANT). This study aimed to determine patients’ anxiety and pain related to WALANT use in outpatient upper limb surgery, and to identify factors that increased the risk of heightened pain experiences.

**Methods:**

This multicentre cross-sectional study conducted between December 2022 and June 2024 utilised the ‘strengthening the reporting of observational studies in epidemiology guidelines’. Patients receiving outpatient upper limb surgery under WALANT were prospectively recruited for participation in an investigator-administered questionnaire of 15 questions regarding their demographics, procedure, anxiety and pain experiences. Patients’ anxiety and pain experiences were summarised, and relationships between demographic or procedural factors and anxiety or pain scores were tested.

**Results:**

In all, 85 patients were included. The most frequent procedures performed were tendon repairs and trigger releases (42% and 12% respectively). Of the total recruited patients, 45% reported moderate to severe anxiety perioperatively. The median visual analogue scale (VAS) score for pain of injection and procedure were 20 mm and 0 mm, respectively. Male patients and isiZulu speaking patients had lower VAS pain scores, while very anxious patients had higher VAS pain scores.

**Conclusion:**

Forty-five per cent of patients who underwent outpatient upper limb surgery under WALANT experienced perioperative anxiety. They did not experience the anaesthetic or surgery as painful on average. Pain scores were related to perioperative anxiety. Future investigation should further explore the relationship between perioperative anxiety and pain.

**Contribution:**

Wide awake local anaesthesia no tourniquet is well tolerated in patients undergoing outpatient upper limb surgery.

## Introduction

To improve the quality of orthopaedic healthcare, researchers are increasingly seeking to understand the experiences of patients, rather than their measured surgical outcomes in isolation.^1^ Patient experience is a broad term generally understood to comprise relational aspects, the patients experience of interactions with their surgeon and other healthcare personnel, and aspects pertaining to their physical care or the perceived management of their healthcare needs.^1^ There is a growing body of evidence to suggest that improving patient experience can positively affect clinical outcomes.^2^ Previous investigation has found that patients with positive experiences are also likely to have a reduction in their postoperative pain experience and be more compliant with rehabilitation.^2,3,4^

A patient’s experience of pain has also been related to anxiety. It has been ascertained that patients with higher preoperative anxiety scores are likely to have worse postoperative pain experiences.^5^ It is therefore concerning that a pilot study investigating elective orthopaedic surgical patients at a South African centre, found the prevalence of preoperative anxiety to be nearly 50%.^6^ Notably, a lack of postoperative support at home and a lower level of education were associated with higher levels of anxiety.^6^ Considering the South African context, where only 31% of adults aged 20 years and older have completed high school, it seems then that patients’ pain, anxiety and perioperative experiences are particularly applicable.^7^

For orthopaedic upper limb surgery, wide awake local anaesthesia no tourniquet (WALANT) has the potential to address a pressing issue relevant to the local context, the ability to perform more cases in an outpatient clinic setting, removing the burden from main operating theatres, access to which is limited.^8^ That is, provided patients’ perioperative experiences of outpatient upper limb surgery are well tolerated, with regard to pain and anxiety. A study investigating the United States of America (US) military setting patients’ experience of WALANT for carpal tunnel, trigger finger and De Quervain’s tenosynovitis releases, found that patients’ anxiety (on a scale where zero denoted no anxiety and 10, intense anxiety) did not exceed 2.8 in any perioperative stage.^9^ Rhee et al. also found there was a significant cost saving by performing the procedure in clinic as opposed to the main operating theatre.^9^ A Malaysian randomised controlled trial comparing patients who received either WALANT or general anaesthesia for distal radius fracture fixation evaluated the difference in patients’ pain and perceived anxiety.^10^ They found no difference in preoperative anxiety or postoperative visual analogue scale (VAS) pain scores between the two groups.^10^ The preliminary findings in South African centres that have adopted WALANT outpatient upper limb surgery have been similar. Naude et al. found the mean VAS score for WALANT administration to be 15 mm, and for the surgery itself 2 mm.^11^ De Buys et al. reported similar VAS scores and 70% of the patients in their cohort reported that the experience was better than expected.^12^

As these previous South African investigations originated from a single province in South Africa, and were conducted at single institutions, this multicentre investigation was developed in a different region, within the public service, to determine if findings are applicable in an alternate South African setting. This investigation aimed to outline patients’ experiences of WALANT for outpatient upper limb surgery, particularly regarding anxiety and pain. Secondary objectives included assessing if any baseline demographic factors, procedural factors or preoperative anxiety scores, placed patients at increased risk for higher pain scores.

## Research methods and design

This multicentre cross-sectional study was conducted during the period December 2022 to June 2024 utilising the ‘strengthening the reporting of observational studies in epidemiology guidelines (STROBE)’ checklist.^13^ All procedures performed were in accordance with the ethical standards of the institutional and/or national research committee, and with the 1964 Helsinki Declaration and its later amendments or comparable ethical standards. None of the five participating public centres have a dedicated outpatient upper limb surgery slate utilising WALANT. As such, outpatient upper limb surgery, and consequently patient enrolment, was performed on an ad-hoc basis according to surgeon and minor theatre availability. Patients were prospectively enrolled and written consent was obtained for all who participated. All adult patients (18 years or older) that were offered WALANT for upper limb surgery, at one of five participating public service centres offering orthopaedic services, with no contra-indications were eligible for inclusion. In addition, patients were required to be conversant in English or isiZulu. The surgeries were performed by specialist orthopaedic surgeons with an interest in hand surgery, or by orthopaedic trainees instructed and overseen by one of the surgeons at one of five participating hospitals during this period. Incomplete questionnaire responses for the nine questions specific to patient pain and anxiety were excluded. Enrolled participants received upper limb surgery according to a predetermined broad set of standards. Standards were agreed upon for location of procedure (minor theatre or office procedure setting where field sterility could be exercised), theatre set-up, equipment requirements, WALANT solution mixing and administration. A detailed outline of the contraindications, standards and a list of upper limb procedures that have previously been effectively performed under WALANT are available as Online Appendix 1.

The questionnaires were administered by the operating surgeon, with or without the aid of a translator, during the perioperative period. Patients were asked to rate their anxiety and fears preoperatively, and postoperatively they were asked to score their pain experience. The questionnaire was available in two media formats. The online format was developed on the REDCap^®^ platform and the questionnaire was completed in real time. For authors with no internet access in the clinical setting, paper-based versions of the questionnaire were printed (available as Online Appendix 1). These questionnaires were then captured to the REDCap^®^ platform in a second sitting, and capturing was checked by an independent investigator. Consent forms were completed in a paper-based format in both cases. The questionnaire comprised 15 questions. The initial six questions pertained to basic patient demographics, the centre at which the procedure was taking place and the nature of the procedure. The next two questions asked the patients to grade their level of anxiety and select as many options as applicable as to the origin of their anxiety or elaborate on the fear if it was not listed. These questions have not been previously validated, but were modelled on those of a previous similar investigation.^9^ Two questions asked patients to score the pain of administration of the WALANT injection and their pain during the operative procedure using a VAS pain score. Thereafter, three questions asked patients how their actual experience related to their preoperative perception, and if they themselves would have a procedure in this manner again or refer a family member or friend to have their procedure in this way. The final two questions related to the duration of the procedure and asked patients to comment on their experience of the procedure.

Previous investigation has determined that the patient accepted symptom state (PASS) for pain measured with VAS is 33 mm and the minimum clinically important difference (MCID) in VAS score is 10 mm.^14^ In this investigation, patients with procedural VAS scores of less than 33 mm were deemed to have tolerated the procedure, and only if significant differences in VAS pain scores equated to 10 mm or more were they considered clinically significant. The sample size necessary to detect a 10 mm difference in VAS scores with a mean of 23.3 mm and standard deviation of 19.4 mm (both of which were established in a previous study) for a power of 0.95 and alpha of 0.05 was calculated to be 51.^14^

The REDCap^®^ data were exported to Microsoft^®^ Excel for Mac (Version 16.86) to collate the descriptive summary statistics and for importing to Jamovi (Version 1.6.23) statistical software for analysis. The primary analysis involved descriptive statistics of patient demographic data, patient anxiety, pain and perception of the experience. The continuous non-parametric variables are expressed as medians with interquartile range (IQR) and range. Categorical variables are expressed in summary with percentages. The pain score for the injection and the procedure was used to calculate an average pain score for each patient. The average pain score was used in a linear regression model, to evaluate the relationships between the pain experienced and preoperative anxiety, type of procedure and other demographic variables. Results of the linear regression are reported with estimates, standard errors (s.e.) and *p*-values. A *p*-value of < 0.05 was deemed statistically significant.

## Results

A total of 85 patients were included in the investigation. No patients were excluded, all questionnaires that were started were completed. Forty-nine per cent of the participants (42 of 85) were male with a median age of 42 years (IQR 20, range 20–67). The majority of patients reported their home language to be isiZulu (68%, 58 of 85), a further 28% (24 of 85) English, and the remaining three spoke Afrikaans, Swahili as well as French and Gujarati, but were conversant in either isiZulu or English. Forty-five per cent of the patients reported at least one co-morbidity (38 of 85). The most frequent procedures performed were tendon repairs in 42% of cases (36 of 85), followed by trigger finger and trigger thumb releases (12%, 10 of 85) and carpal tunnel releases (9%, 8 of 85). Table 1 summarises the patient demographics and procedural information. The median surgical duration for participants was 43 min (IQR 35, range 5–150 min).

**TABLE 1:** Descriptive statistics for the baseline demographics and procedures of the patients (*N* = 85).

Characteristic	*n*	%	Median	IQR	Range
**Demographics**					
Age (years)	85	100.0	42	20	20–67
Gender					
Female	43	50.6	-	-	-
Male	42	49.4	-	-	-
Home language					
Zulu	58	68.2	-	-	-
English	24	28.2	-	-	-
Afrikaans	1	1.2	-	-	-
French and Swahili	1	1.2	-	-	-
Gujarati	1	1.2	-	-	-
Co-morbidities					
Yes	38	44.7	-	-	-
No	47	55.3	-	-	-
**Procedural details**					
Hospitals					
1	43	50.6	-	-	-
2	6	7.1	-	-	-
3	21	24.7	-	-	-
4	14	16.5	-	-	-
5	1	1.2	-	-	-
Duration (minutes)	85	100.0	43	35	5–150
Procedures					
Carpal tunnel	8	9.4	-	-	-
Digital amputations	2	2.4	-	-	-
Tendon procedure (repairs)	36	42.4	-	-	-
Trigger finger or thumb	10	11.8	-	-	-
Excision biopsy ± FTSG graft	10	11.8	-	-	-
De Quervain’s disease	6	7.1	-	-	-
Dupuytren’s release	3	3.5	-	-	-
Digital nerve neurolysis	2	2.4	-	-	-
Foreign body removal	2	2.4	-	-	-
Open reduction MCPJ dislocation	1	1.2	-	-	-
MCPJ release	1	1.2	-	-	-
Single tendon transfer	2	2.4	-	-	-
Ulnar nerve transposition	1	1.2	-	-	-
Volar ganglion excision	1	1.2	-	-	-

Note: Continuous variables expressed as medians with IQR and range. Categorical variables expressed with counts and percentages of total.

IQR, interquartile range; FTSG, Full thickness skin graft; MCPJ, metacarpophalangeal joint.

Regarding patients’ anxiety (Table 2), less than half the patients reported moderate to severe anxiety (45%, 38 of 85), that is a score on a Likert scale from 1 to 5 of 3 or more. Most patients reported that this anxiety was related to the fear that they would experience pain during the procedure (52%, 44 of 85). Fear of witnessing the procedure or hearing the sounds of the procedure were less contributary to their anxiety, scoring 19% (16 of 85) and 13% (11 of 85), respectively. Several of the patients reported being fearful for a reason other than those listed (26%, 22 of 85), and they reported a fear of the unknown, because they had never undergone surgery before 8% (7 of 85), or the fear that their hand or limb would not work effectively after the operation or that the surgery would not be a success 12% (10 of 85).

**TABLE 2:** Descriptive and inferential statistics for anxiety, pain and patient experience.

Characteristic	*n*	%	Median	IQR	Range	Estimate	s.e.	*p*-value
**Anxiety prior to procedure**								
1 (none)	25	29.4	-	-	-	-	-	-
2 (mild)	22	25.9	-	-	-	-	-	-
3 (moderate)	19	22.4	-	-	-	-	-	-
4 (very)	6	7.1	-	-	-	-	-	-
5 (severe)	13	15.3	-	-	-	-	-	-
Fear of pain despite anaesthetic	44	51.8	-	-	-	-	-	-
Fear of having to witness the procedure	16	18.8	-	-	-	-	-	-
Fear of hearing sounds during the procedure	11	12.9	-	-	-	-	-	-
Someone else had a bad experience with local anaesthesia	4	4.7	-	-	-	-	-	-
I have had an unsuccessful local anaesthesia	3	3.5	-	-	-	-	-	-
Other fears not listed (22 of 85)	22	25.9	-	-	-	-	-	-
Fear of unknown	7	8.2	-	-	-	-	-	-
Loss of function or fear procedure will not succeed	10	11.8	-	-	-	-	-	-
Fear of needles	2	2.4	-	-	-	-	-	-
Fear of post-operative pain	1	1.2	-	-	-	-	-	-
**Pain (mm)**								
VAS pain score for injection	85	100.0	20	23	0–82	-	-	-
VAS pain score for operation	85	100.0	0	7	0–87	-	-	-
Median VAS pain score for injection and procedure	85	100.0	10	15	0–45	-	-	-
**Patient experience**								
Procedure as expected								
As expected	5	5.9	-	-	-	-	-	-
Better than expected	80	94.1	-	-	-	-	-	-
Worse than expected	0	0.0	-	-	-	-	-	-
Would you have WALANT again?								
Unsure	1	1.2	-	-	-	-	-	-
Yes	84	98.8	-	-	-	-	-	-
No	0	0.0	-	-	-	-	-	-
Would you recommend WALANT?								
Unsure	1	1.2	-	-	-	-	-	-
Yes	84	98.8	-	-	-	-	-	-
No	0	0.0	-	-	-	-	-	-
**Factors with relationships to total VAS for pain**								
Male gender	-	-	-	-	-	−6.53	2.45	0.009
Zulu home language	-	-	-	-	-	−8.13	2.73	0.004
Very anxious	-	-	-	-	-	10.30	5.19	0.050

Note: Continuous variables expressed as medians with IQR and range. Categorical variables expressed with counts and percentages of total.

IQR, interquartile range; VAS, visual analogue scale 0 mm – 100 mm; mm, millimetres; WALANT, wide awake local anaesthesia no tourniquet; s.e., standard error.

The median VAS score for pain of the injection was 20 mm (IQR 23 mm, range 0 mm-82 mm) and the median VAS score for the pain experienced during the operation was 0 mm (IQR 7 mm, range 0 mm-87 mm). The combined average pain score (for both the injection and the operation) was 10 mm (IQR 15 mm, range 0 mm-45 mm). Following the procedure most patients (94%, 80 of 85) reported that the procedure was better than expected, would have a procedure with the same anaesthetic technique again (99%, 84 of 85) and would recommend this technique to their family and friends (99%, 84 of 85).

Regarding the relationships of demographics and procedural factors to anxiety and pain, no baseline demographic factors or procedural factors were found to be related to the anxiety reported by patients, or were underpowered to detect significance. Several demographic factors and reported anxiety were related to patients’ pain experiences. Female patients had an estimated VAS pain score of 6.53 mm higher than their male counterparts (s.e. 2.45, *p* 0.009). Patients who reported isiZulu as their home language had an estimated VAS pain score of 8.13 mm lower than the English speaking patients (s.e. 2.73, *p* 0.004). Anxiety scores were also related to pain. Very anxious patients (with a score of 4 on the anxiety scale) had an estimated VAS pain score of 10.30 mm higher (s.e. 5.19, *p* 0.05) than patients in the moderately anxious group (score of 3). Table 2 highlights the factors significantly associated with patients’ pain experiences.

## Discussion

This investigation aimed to delineate patients’ experiences of WALANT outpatient upper limb surgery, with particular focus on their anxiety and how it relates to their pain. Just under half of the patients reported moderate to severe anxiety preoperatively. Previous investigation regarding anxiety in patients undergoing day-case procedures has identified certain patient demographic risk factors, triggers for anxiety and anaesthetic risk factors. Regarding demographics, both Mitchell and Caddick et al. determined that younger patients and female patients are more likely to experience preoperative anxiety.^15,16^ We did not find an association to anxiety levels for age or gender. A contributory factor for anxiety, proposed by Mitchell, is a lack of information or fear related to the unknowns of the anaesthetic and surgery.^15^ Mitchell found that 60% of the patients in his study required a high level of information, and also expressed the need for this information between 1 and 4 weeks prior to surgery.^15^ Nine per cent of the patients in the present study expressed anxiety related to the unknown. Based on the findings of Mitchell, perhaps the fact that patients in the present investigation were only given information immediately prior to the procedure (and that this information was weighted towards the research investigation and only secondarily covered the anaesthesia and the procedure) could have contributed to their anxiety. However, irrespective of the timing of counselling, it does highlight the importance of attention to information sharing with patients if you are to adopt WALANT in practice. As foreseen, the majority of patients were also anxious about the potential of experiencing pain (51%), seeing the operation take place (19%) or hearing the sounds of the surgery (14%). This sentiment has been echoed in several prior investigations.^15^ Interestingly though, one investigation highlighted that in conscious patients, not only the potential pain experience, but conversation from the surgeon that included words such as ‘scalpel’ or ‘knife’ were also anxiety provoking.^16^

The original intent of recording the home language of patients for the present study concerned obtaining a veracious consent. But, considering the influence that the use of words played on patients anxiety that the aforementioned investigation highlighted, it would have been interesting to see if this relationship occurred in the present investigation. While we were unable to detect a relationship between home language and anxiety, we did find that home language was related to the pain reported by our patient cohort. Previous work in Saudi Arabia investigating patients recovering from cardiac surgery, showed that when nursing care was provided by a non-Arabic-speaking nurse, more anxiety was reported compared to patients cared for by Arabic-speaking nurses.^17^ This does not conclusively show that a language barrier alone is responsible for increased anxiety; however, as the authors argued, language barriers do have the potential to influence patients experience of care.

The anaesthetic type has also been shown to be related to patient anxiety experience.^15^ Although local anaesthetic procedures provoke anxiety, it would seem that patients have more anxiety related to general anaesthesia.^15^ Perhaps then, anxiety related to anaesthesia and surgery is unavoidable and, day case WALANT procedures are worthwhile adopting. Previous work has also shown WALANT to be experienced as convenient by patients.^9^ The convenience potentially superseding the initial anxiety of being awake for the procedure. Provided that patients tolerate the pain of administering the local anaesthesia.

The patients in the present investigation experienced pain with a median VAS score of 20 mm, for administration of the WALANT injection, and 0 mm for the procedure itself. This means that on average, both the administration of the injection and the procedure itself, are below the PASS for pain measured with VAS (33 mm), which signifies that WALANT is generally well tolerated by the patients of this investigation.^14^ Gender, home language and anxiety were related to pain scores. Female patients were found to have higher VAS pain scores than men. Although the difference in VAS pain scores were statistically significant, they came in below the 10 mm value for MCID in VAS pain scores.

Low pain scores following utilisation of the WALANT technique have been reported in several previous investigations, both locally and abroad.^9,11,12,18^ Considering the patients’ pain experiences, the finding that 99% of patients would undergo WALANT for similar procedures in the future if indicated, and 99% would also recommend outpatient upper limb surgery with WALANT to family and friends, is consistent. A finding corroborated by Pina et al. who similarly reported that 98%, and 84% of the group tested by Rhee et al., of patients would be willing to have a WALANT anaesthetic in the future if indicated.^9,18^

Yet, with these positive pain experiences and increasing adoption of the technique in hand surgery, up to 40% of hand surgeons have not yet incorporated it into their practice.^19,20^ The most common barriers cited include the now disproven belief regarding the risk of extremity necrosis with local epinephrine (adrenalin) administration, the consideration of patients’ anxiety and pain related to local administration and being awake for the procedure (examined and challenged by the present investigation), and institutional barriers (such as regulations and funding of office-based procedures and staffing).^19^ It will be important to challenge these barriers if widespread utility is to be appreciated.

The were several limitations of this study. This is a smaller group of patients than similarly designed investigations, and while this may have limited full exploration of factors associated with pain and anxiety, it was sufficiently powered to demonstrate some strong associations. Also, some factors previously found to influence patients’ perioperative anxiety and pain such as level of education and home support systems were not included in the investigation and could have provided a more holistic understanding of the patients’ anxiety and pain experiences. Finally, the implications of patients pain and anxiety experiences which have previously been suggested to change compliance to rehabilitation and the surgical outcomes were not addressed here, but would be important to explore in future investigation.

## Conclusion

Of patients who undergo outpatient upper limb surgery under WALANT, 45% experience moderate to severe preoperative anxiety. They do not experience the anaesthetic or surgical procedure as painful on average. Pain scores are related to perioperative anxiety. Nearly all patients are willing to undergo a WALANT anaesthetic again if indicated and would recommend the technique.
